# Impact of Culture-Positive Preservation Fluid on Early Morbidity and Mortality After Lung Transplantation

**DOI:** 10.3389/ti.2023.10826

**Published:** 2023-02-08

**Authors:** Alexy Tran-Dinh, Imane Tir, Sébastien Tanaka, Enora Atchade, Brice Lortat-Jacob, Sylvain Jean-Baptiste, Nathalie Zappella, Sandrine Boudinet, Yves Castier, Hervé Mal, Pierre Mordant, Iannis Ben Abdallah, Vincent Bunel, Jonathan Messika, Laurence Armand-Lefèvre, Nathalie Grall, Philippe Montravers

**Affiliations:** ^1^ Université Paris Cité, AP-HP, Hôpital Bichat Claude Bernard, Département d'Anesthésie-Réanimation, Paris, France; ^2^ INSERM UMR 1148 LVTS, Université de Paris, Paris, France; ^3^ Réunion Island University, INSERM U1188 Diabetes Atherothrombosis Réunion Indian Ocean (DéTROI), CYROI Plateform, Saint-Denis de la Réunion, France; ^4^ Université Paris Cité, AP-HP, Hôpital Bichat Claude Bernard, Service de Chirurgie Vasculaire, Thoracique et Transplantation Pulmonaire, Paris, France; ^5^ INSERM UMR 1152 PHERE, Université de Paris, Paris, France; ^6^ Université Paris Cité, AP-HP, Hôpital Bichat Claude Bernard, Pneumologie B et Transplantation Pulmonaire, Paris, France; ^7^ Paris Transplant Group, Paris, France; ^8^ Université Paris Cité, AP-HP, Hôpital Bichat Claude Bernard, Service de Bactériologie, Paris, France; ^9^ INSERM UMR 1137 IAME, Université de Paris, Paris, France

**Keywords:** lung transplantation, survival, preservation fluid, antibiotic prophylaxis, ICU morbidity

## Abstract

The prevalence, risk factors and outcomes associated with culture-positive preservation fluid (PF) after lung transplantation (LT) are unknown. From January 2015 to December 2020, the microbiologic analyses of PF used to store the cold ischaemia-placed lung graft(s) of 271 lung transplant patients were retrospectively studied. Culture-positive PF was defined as the growth of any microorganism. Eighty-three (30.6%) patients were transplanted with lung grafts stored in a culture-positive PF. One-third of culture-positive PF were polymicrobial. Staphylococcus aureus and Escherichia coli were the most frequently isolated microorganisms. No risk factors for culture-positive PF based on donor characteristics were identified. Forty (40/83; 48.2%) patients had postoperative pneumonia on Day 0 and 2 (2/83; 2.4%) patients had pleural empyema with at least one identical bacteria isolated in culture-positive PF. The 30-day survival rate was lower for patients with culture-positive PF compared with patients with culture-negative PF (85.5% vs. 94.7%, *p* = 0.01). Culture-positive PF has a high prevalence and may decrease lung transplant recipient survival. Further studies are required to confirm these results and improve understanding of the pathogenesis of culture-positive PF and their management.

## Graphical Abstract

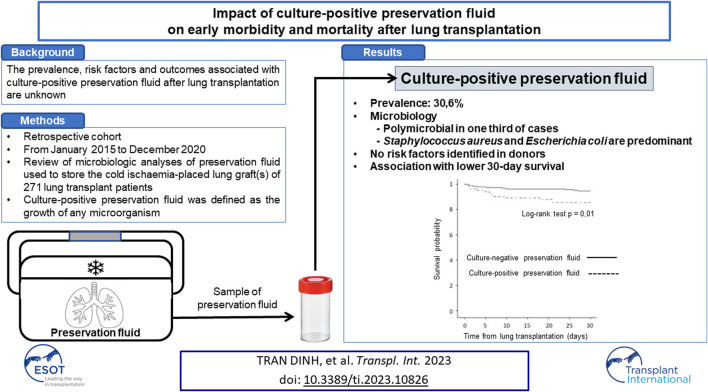

## Introduction

Lung transplantation (LT) is the final resort therapy for patients with end-stage lung disease (1). Infections strongly decrease recipient survival, accounting for 17% and 33% of deaths at 30 days and 1 year, respectively (2). Among the various potential sources of posttransplant infections, donor-to-host transmission of infection in solid organ transplant is a life-threatening early complication (3–5). In a prospective study assessing 211 donors contributing to 292 transplant procedures, lung was the most likely to be performed with an infected donor (15%), although only one donor-transmitted infection occurred (6).

Investigating early lung graft infection may include peri-transplant microbiological culture of donor and recipient respiratory specimens as well as organ preservation fluid (PF) (7). Culture-positive PF may indicate graft infection, contamination during graft procurement or colonization by passage of the causative microorganisms from the organ into the storage fluid during cold ischaemic time. However, there is no recommendation for its evaluation and use to guide antibiotic therapy. A recent systematic review and meta-analysis among solid organ transplants observed an overall incidence of culture-positive PF and PF-related infections of 37% and 10%, respectively, and mortality rates among PF-related infections of 35% (8). However, specific data in LT remain very limited (5, 9).

To address this issue, we sought to describe 1) the prevalence of culture-positive PF and PF-related postoperative pneumonia and 2) risk factors and outcomes associated with culture-positive PF in LT. We also evaluated the impact of the adequacy between the peri-transplant antibiotic prophylaxis and the susceptibility of microorganisms isolated from PF on recipient outcomes.

## Materials and Methods

### Study Design

We conducted a retrospective single-centre study that included all consecutive patients who underwent LT between January 2015 and December 2020. Retransplantations and *ex vivo* lung perfusion procedures were not included.

We analysed all available microbiological cultures of PF, donor respiratory specimens performed before lung procurement, and recipient respiratory specimens collected during postoperative ICU admission. The study was approved by the ethics committee CEERB Paris Nord, which waived the need for signed informed consent (Institutional Review Board -IRB 00006477- Université Paris Cité, AP-HP.Nord).

Donor lung procurement was performed identically for bilateral and single LT. Lungs were procured “*en bloc*” with the trachea immediately stapled to avoid subsequent PF contamination, stored in a bag and immersed in PF (Perfadex^®^, XVIVO, Goteborg, Sweden). The bag was surrounded by ice to maintain the temperature at 4°C during the cold ischaemia time for transport to our centre. Lung separation was performed on a back table upon arrival at our centre after removal from the bag containing the PF. A sample of PF was taken and sent for microbiological culture (10).

### Microbiological Features and Definitions

“PF samples were systematically collected in sterile by the surgeon during graft preparation on the back table during pneumonectomy of the native lung(s), and immediately sent to the bacteriology and mycology laboratories. Samples were inoculated all day every day onto routine agar plates (100 µL per plate), which included trypticase soy agar supplemented with 5% horse blood, Columbia sheep blood agar containing nalidixic acid and colistin and chocolate agar supplemented with PolyVitex for isolation of fastidious bacteria. The plates were incubated for 48 h at 35 ± 2°C under aerobic and anaerobic conditions. The limit of detection was 10^2^ UFC/mL. All culture media were controlled weekly by the culture of ATCC strains according to applicable standards. All the different morphotypes of colonies that grew on the different plates were identified at the species level by matrix-assisted laser desorption ionization-time of flight mass spectrometry (MALDI-TOF MS) Microflex LT Biotyper (Bruker Daltonics, Bremen, Germany). Bacterial susceptibility to antibiotics was determined using the disk-diffusion method according to EUCAST (European Committee on Antimicrobial Susceptibility Testing) for all bacteria isolated. Culture-positive PF was defined as the growth of any microorganism.”

High- and low-risk microorganisms were defined as described previously (11). High-risk microorganisms included Gram-negative bacilli (GNB), *Staphylococcus aureus*, *β-haemolytic Streptococcus* spp., *Streptococcus pneumoniae*, *Enterococcus* spp., *Bacteroides* spp., and *Candida* spp. Low-risk microorganisms included *coagulase-negative Staphylococcus* spp., *Corynebacterium* spp., and α-haemolytic *Streptococcus* spp. In our local policy, patients with culture-positive PF were treated for 7 days with appropriate antibiotic therapy, regardless of the results of respiratory samples on Day 0, because of the theoretical risk of pleural empyema.

Donor respiratory samples taken just prior to lung procurement by the thoracic surgeon of our centre were microbiologically analysed at the donor centre. The results were retrieved *via* the Agence de la Biomédecine, a national public agency in charge of coordination of organ, tissue and cell procurement and transplantation, as well as in the fields of human reproduction, embryology and genetics (12). There was no microbiological analysis of the donor lung just before its transplantation into the recipient.

Upon ICU admission after LT, bronchoalveolar lavage (BAL) respiratory specimens were taken from the recipient and analysed for microbiological culture. Postoperative bacterial pneumonia on Day 0 was defined as in the recommendations for cardiothoracic transplant patients (13). A diagnosis of pneumonia was established when clinical, biological, radiographic and microbiological criteria were met. Clinical, biological and radiographic criteria were fever (temperature >38°C), purulent secretions, gas exchange degradation, elevated white blood cell count, and chest imaging revealing a new or progressive alveolar or interstitial that could not be explained by any other noninfectious cause. Microbiological criteria was a positive bacterial culture at the threshold of infection of a bronchoalveolar lavage (BAL) performed at postoperative ICU admission (14, 15). Patients with pneumonia were treated for 7 days with appropriate antibiotic therapy, and were considered cured if signs of infection resolved (improvement in clinical signs, haematosis and radiological abnormalities).

### Data Collection

Donor characteristics, including age, sex, smoking status, cause of death, duration of mechanical ventilation, and PaO_2_/FiO_2_ ratio at the time of lung procurement, were collected.

Demographic and preoperative characteristics of recipients were recorded, including age, sex, body mass index (BMI), primary diagnosis of chronic pulmonary disease, Cytomegalovirus mismatch (Donor+/Recipient-), past medical history of diabetes and ischaemic heart disease with angioplasty and/or coronary stent, high-emergency LT, extracorporeal membrane oxygenation (ECMO) as bridge to transplant and mean pulmonary arterial pressure (mPAP) measured by a right heart catheterization at listing. High emergency LT is a national prioritization system for the most severe patients with fibrosis, cystic fibrosis or pulmonary hypertension that was introduced in France in 2007 (16).

Intraoperative characteristics were collected, including type of LT (i.e., single or bilateral), maximum graft cold ischaemia time, intraoperative blood transfusion >2 packed red blood cells (PRBC) and intraoperative ECMO.

Lung graft complications were also documented, including grade 3 primary graft dysfunction (PGD) as defined by the ISHLT consensus (17), acute cellular rejection confirmed by histopathological evidence after transbronchial lung biopsies or considered and treated as if the risk of biopsy outweighed the expected benefit (18), and definite, probable or possible antibody-mediated rejection according to Levine et al. (19) with the need for plasmapheresis.

We recorded patient outcomes, including ICU stay characteristics (simplified acute physiology score II (SAPS II) and sequential organ failure assessment (SOFA) score at admission, acute kidney injury stage 3 of KDIGO (Kidney Disease: Improving Global Outcomes), renal replacement therapy, duration of mechanical ventilation, duration of norepinephrine support, ECMO in ICU, tracheostomy, ICU length of stay, chronic lung graft dysfunction and mortality rates at 30 days, 1, 3, and 5 years.

### Perioperative Management

Surgical transplantation procedures and perioperative care, including postoperative management, were standardized for all patients according to our local protocol already published elsewhere (20). The immunosuppressive regimen included mycophenolate mofetil, corticosteroids and tacrolimus. There was no induction therapy.

Perioperative antibiotic prophylaxis was defined by the antibiotic regimen started intraoperatively. It was considered appropriate towards the PF culture if it was effective against all bacteria isolated in the PF after susceptibility testing. Perioperative antibiotic prophylaxis was cefazolin, as it was recommended in “Clinical practice guidelines for antimicrobial prophylaxis in surgery” (21). Cefazolin was substituted and tailored according to the known colonisation of the donor and recipient. Perioperative antibiotic prophylaxis was systematically administered intraoperatively and continued 48 h after surgery, as recommended (22). During the immediate postoperative period, antibiotic therapy was adapted to microbiological cultures obtained from bronchoalveolar lavage (BAL) performed upon postoperative ICU admission and from PF. If BAL and PF cultures were negative without evidence of infection, antibiotic prophylaxis was stopped after 48 h.

### Statistical Analysis

Baseline characteristics within each group were described with numbers and percentages for qualitative variables and medians and interquartile ranges for quantitative variables.

Thirty-day and 1-year survival rates were assessed between patients with culture-positive PF and culture-negative PF and between patients who received or did not receive an appropriate peri-operative antibiotic prophylaxis for culture-positive PF. The probability of all-cause death was estimated using the Kaplan-Meier method and compared using the log-rank test.

Donor risk factors for culture-positive PF were assessed by univariate analysis, and unadjusted odds ratios (ORs) and 95% CIs were calculated.

All reported *p* values were two-sided, and the level of statistical significance was specified *a priori* as less than 0.05. Statistical analysis and data management were performed using BM SPSS Statistics version 20 (IBM Corp., Armonk, NY, United States).

## Results

Two hundred seventy-one patients were transplanted with one or two lung grafts procured from 271 donors between January 2015 and December 2020. The median age of recipients at the time of LT was 57 [50–62] years. Primary diagnoses were mainly interstitial lung disease (48.3%) and COPD (36.2%). Double LT represented 67.9% of the procedures (Table 1).

**TABLE 1:** Recipient demographics and intraoperative characteristics.

	All patients (*n* = 271)	Culture-positive PF (*n* = 83)	Culture-negative PF (*n* = 188)	OR [95% CI], *p* value
Recipient demographics and comorbidities				
Age, years	57 [50–62]	57 [50–62]	51 [56–62]	1.01 [0.99–1.03], *p* = 0.37
Female sex	97 (35.8)	56 (67.5)	118 (62.8)	1.23 [0.71–2.12], *p* = 0.49
BMI, kg/m^2^	24 [20–27]	24 [20–28]	24 [20–27]	1.01 [0.94–1.07], *p* = 0.77
Aetiology				
COPD	98 (36.2)	33 (39.8)	65 (34.6)	1.25 [0.73–2.13], *p* = 0.41
ILD	131 (48.3)	35 (42.2)	96 (51.1)	0.70 [0.42–1.12], *p* = 0.18
Others	43 (16)	16 (19.8)	27 (14.4)	1.46 [0.74–2.89], *p* = 0.28
Coronary angioplasty and/or stent	11 (4.1)	1 (1.2)	10 (5.3)	0.22 [0.03–1.72], *p* = 0.11
Diabetes	28 (10.3)	8 (9.6)	20 (10.6)	0.90 [0.78–2.13], *p* = 0.80
mPAP, mmHg	25 [20–30]	25 [20–30]	25 [21–30]	0.74 [0.96–1.03], *p* = 0.99
CMV mismatch	56 (20.7)	16 (19.3)	40 (21.4)	0.88 [0.46–1.68], *p* = 0.69
ECMO as bridge-to-transplant	20 (7.4)	6 (7.2)	14 (7.4)	0.97 [0.36–2.62], *p* = 0.95
High-emergency LT	49 (18.1)	15 (18.1)	34 (18.1)	1.0 [0.51–1.96], *p* = 1
Lung transplant surgery				
Type of LT				0.97 [0.56–1.69], *p* = 0.92
Single LT	87 (32.1)	27 (32.5)	60 (31.9)	
Double LT	184 (67.9)	56 (67.5)	128 (68.1)	
Maximum graft ischaemic time, min	330 [270–400]	330 [270–400]	333 [270–400]	1.0 [0.99–1.0], *p* = 0.88
Intraoperative ECMO	190 (70.1)	59 (71.1)	131 (69.7)	1.07 [0.61–1.89], *p* = 0.82
Transfusion ≥3 PRBC	128 (47.6)	40 (48.2)	88 (47.3)	1.04 [0.62–1.74], *p* = 0.89

Quantitative variables are expressed as medians and interquartile ranges. Qualitative variables are expressed as numbers and percentages.

Abbreviations: PF, preservation fluid; BMI, body mass index; COPD, chronic obstructive pulmonary disease; ILD, interstitial lung disease; PAP, pulmonary arterial pressure; CMV, cytomegalovirus; ECMO, extracorporeal membrane oxygenation; LT, lung transplantation; PRBC, packed red blood cell.

### Prevalence of Culture-Positive PF and Microbiological Components

Eighty-three (30.6%) patients were transplanted with lung grafts stored in a culture-positive PF. Microorganisms isolated in PF are presented in Table 2. Twenty-seven (27/83 = 32.5%) PFs were polymicrobial, and 73 (73/83; 88%) were positive for at least one “high-risk” microorganism. *Staphylococcus aureus* and *Escherichia coli* were the most frequently isolated microorganisms. Four (4/83; 4.8%) PF were positive for at least one fungus. None were positive for extended-spectrum beta-lactamase-producing Enterobacteriaceae or multidrug-resistant bacteria.

**TABLE 2:** Microorganisms isolated from culture-positive PF.

Microorganisms (*n* = 108)	(*n*)
High-risk pathogens (*n* = 91; 84.3%)	
Bacterial species (*n* = 86; 79.6%)	
Gram-negative bacilli (*n* = 51; 47.2%)	
*Escherichia coli*	13
*Enterobacter cloacae*	6
*Klebsiella pneumoniae*	5
*Pseudomonas aeruginosa*	4
*Klebsiella aerogenes*	4
*Serratia marcensens*	4
*Klebsiella oxytoca*	3
*Citrobacter koseri*	3
*Haemophilus influenzae*	3
*Hafnia alvei*	2
*Proteus mirabilis*	2
*Serratia ureilytica*	1
*Acinetobacter pitii*	1
Gram-positive cocci (*n* = 35; 32.4%)	
*Staphylococcus aureus*	33
*Streptococcus pneumoniae*	2
Fungal species (*n* = 5; 4.6%)	
*Candida albicans*	2
*Candida glabrata*	1
*Candida parapsilosis*	1
*Candida krusei*	1
Low-risk pathogens (*n* = 17; 15.7%)	
*Oropharyngeal flora* *^a^*	8
*Branhamella catarrhalis*	2
*Streptococcus anginosus*	2
*Staphylococcus epidermidis*	2
*Streptococcus oralis*	1
*Corynebacterium striatum*	1
*Corynebacterium propinquum*	1

^a^ Bacterial species composing the oropharyngeal flora are a-hemolytic streptococci excepted *Streptococcus pneumoniae*, *Haemophilus* spp. excepted *Haemophilus influenzae*, *Neisseria* spp. excepted *Neisseria meningitidis* and *Neisseria gonorrhoeae* and *Rothia mucilaginosa*.

Antibiotic prophylaxis other than cefazolin (*n* = 183, 67.5%) were amoxicillin/clavulanic acid (*n* = 39, 14.4%), cefepime (*n* = 28, 10.3%), ceftazidime (*n* = 6, 2.2%), piperacillin/tazobactam (*n* = 7, 2.6%), cefotaxime (*n* = 5, 1.8%), carbapenem (*n* = 3, 1.1%) and linezolid (*n* = 4, 1.5%).

### Risk Factors for Culture-Positive PF

We did not identify risk factors for culture-positive PF from donor characteristics or preoperative and intraoperative recipient characteristics (Tables 1, 3).

**TABLE 3:** Donor risk factors associated with culture-positive PF.

Donor characteristics	All patients (*n* = 271)	Culture-positive PF (*n* = 83)	Culture-negative PF (*n* = 188)	OR [95% CI], *p* value
Age, years	53 [41–61]	51 [39–61]	53 [42–62]	0.97 [0.98–1.01], *p* = 0.68
Female sex	121 (44.6)	34 (41)	87 (46.3)	1.24 [0.74–2.09], *p* = 0.42
Active smoking	100 (36.9)	35 (42.2)	65 (34.6)	1.38 [0.81–2.34], *p* = 0.23
Cerebral cause of death	214 (79)	62 (74.7)	152 (80.9)	0.70 [0.38–1.29], *p* = 0.25
Duration of mechanical ventilation, days				1.02 [0.94–1.10], *p* = 0.65
PaO_2_/FiO_2_, mmHg	398 [343–459]	383 [331–446]	400 [347–463]	0.98 [0.99–1.0], *p* = 0.22

Quantitative variables are expressed as medians and interquartile ranges. Qualitative variables are expressed as numbers and percentages.

Abbreviations: PF, preservation fluid.

#### Respiratory Samples From Donor Lung

Two hundred and twenty (220/272 = 81.2%) donors had a respiratory sample before lung procurement. Ninety-one (91/220 = 41.4%) had culture-positive respiratory samples. Details of the bacteria isolated from donor respiratory samples are presented in the Supplementary Table S1. Donors had no pneumonia or pneumonia controlled by antibiotic therapy without infiltrates on the CT scan prior to organ procurement.

Among the 83 recipients grafted with culture-positive PF, 40 donors had positive microbiological cultures of respiratory specimens, 20 donors had negative cultures, and 23 donors did not have available respiratory specimens. Twenty-eight (28/40; 70%) recipients had at least one identical microorganism documented in both the PF and the donor respiratory samples.

### Postoperative Outcomes

#### Mortality, ICU morbidity and chronic lung graft dysfunction

**FIGURE 1:**
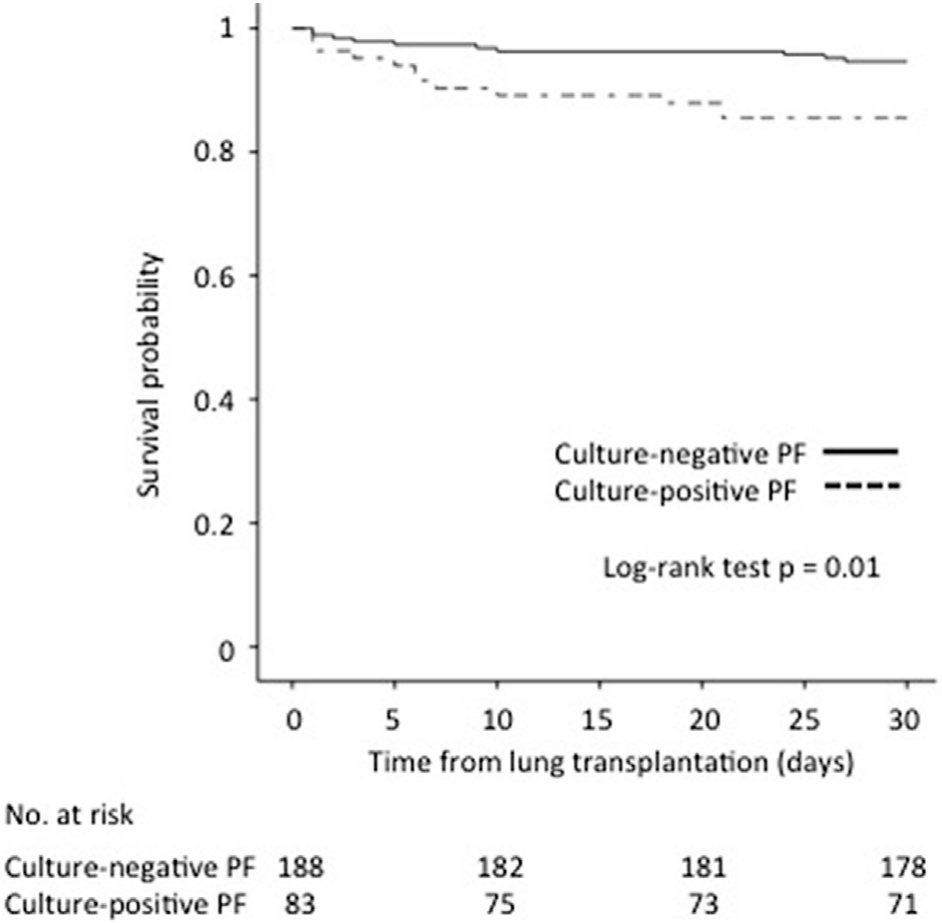
Impact of culture-positive PF on 30-day survival after lung transplantation.

The 30-day survival rate was significantly lower for patients with culture-positive PF compared with patients with culture-negative PF (85.5% vs. 94.7%, *p* = 0.01) (Figure 1).

Survival rates at 1, 3, and 5 years for patients with culture-positive PF compared with patients with culture-negative PF were 68.7% vs. 78.7% (*p* = −0.06), 48.5% vs. 62% (*p* = 0.06) and 32.4% vs. 52.7% (*p* = 0.04), respectively.

Deaths at 30 days (*n* = 22) were due to haemorrhagic shock (*n* = 10, 45.5%), septic shock (*n* = 6, 27.3%), primary graft dysfunction (*n* = 2, 9%) or others (*n* = 4, 18.2%). In the group of patients with culture-positive PF who died within 30 days (*n* = 12/83, 14.5%), the causes of death were haemorrhagic shock (*n* = 6, 50%), septic shock (*n* = 4, 33.3%, two of which were related to pneumonia with the same germ identified in the PF), primary graft dysfunction (*n* = 1, 8.3%) and other (*n* = 1, 8.3%). In the group of patients with culture-negative PF who died within 30 days (*n* = 10/188, 5.3%), the causes of death were haemorrhagic shock (*n* = 4, 40%), septic shock (*n* = 2, 20%), primary graft dysfunction (*n* = 1, 10%) and others (*n* = 3, 30%).

Patients with culture-positive PF had higher SAPS II scores on postoperative ICU admission, had more AKI and required more RRT and ECMO during their ICU stay (Table 3).

The occurrence of chronic lung graft dysfunction was similar between recipients with culture-positive PF and culture-negative PF (27.9% vs. 28.8%, *p* = 0.89).

#### Postoperative Pneumonia on Day 0 and Pleural Empyema

One hundred and twenty-one (121/271 = 44.6%) recipients had postoperative pneumonia on Day 0. The 30-day survival rate between recipients who had postoperative pneumonia on Day 0 compared to those who did not was similar (94% vs. 89.3%, *p* = 0.18). The overall number of pneumonia cases during the ICU stay was similar in recipients with and without culture-positive PF (Table 4). Bacteria isolated from pneumonia occurring during the ICU stay of patients transplanted with lung graft(s) stored in culture-positive or culture-negative PF are presented in the Supplementary Table S2.

**TABLE 4:** Outcomes associated with culture-positive PF.

Outcomes	All patients (*n* = 271)	Culture-positive PF (*n* = 83)	Culture-negative PF (*n* = 188)	OR [95% CI], *p* value
Postoperative ICU stay				
Postoperative SAPS II	43 [38–50]	46 [38–53]	43 [38–50]	1.03 [1.01–1.05], *p* = 0.01
Postoperative SOFA score	7 [6–9]	8 [6–10]	7 [6–9]	1.07 [0.96–1.19], *p* = 0.23
Stage 3 AKI of KDIGO	39 (14.4)	19 (22.9)	20 (10.7)	2.48 [1.24–4.95], *p* = 0.009
Renal replacement therapy	31 (11.5)	16 (19.3)	15 (8.1)	2.72 [1.28–5.82], *p* = 0.008
Duration of mechanical ventilation, days	3 [1–19]	4 [1–19]	3 [1–14]	1.0 [0.99–1.01], *p* = 0.59
Duration of norepinephrine, days	2 [1–4]	2 [1–4]	2 [1–4]	1.01 [0.96–1.06], *p* = 0.65
ECMO in ICU	77 (28.5)	31 (37.3)	46 (24.6)	1.83 [1.05–3.19], *p* = 0.03
Tracheotomy	66 (24.6)	23 (28.4)	43 (23)	1.33 [0.74–2.40], *p* = 0.35
Length of ICU stay, days	17 [10–33]	16 [10–32]	17 [11–33]	1.0 [0.96–1.01], *p* = 0.47
Lung graft complications				
Grade 3 primary graft dysfunction	48 (17.8)	16 (19.5)	32 (17.1)	1.17 [0.60–2.29], *p* = 0.73
Postoperative pneumonia on Day 0	121 (44.6%)	51 (61.4)	70 (37.2)	2.68 [1.58–4.57], *p* < 0.001
Number of pneumonia cases during ICU stay	1 [0–2]	1 [0–2]	1 [0–2]	1.05 [0.86–1.28], *p* = 0.64
Acute antibody-mediated rejection	53 (19.7)	16 (19.5)	37 (19.8)	0.98 [0.51–1.89], *p* = 0.98
Acute cellular rejection	62 (23.1)	17 (21)	45 (24.1)	0.84 [0.45–1.58], *p* = 0.58

Quantitative variables are expressed as medians and interquartile ranges. Qualitative variables are expressed as numbers and percentages.

Abbreviations: PF, preservation fluid; ICU, intensive care unit; SAPS II, simplified acute physiology score II; SOFA, sequential organ failure assessment; AKI, acute kidney injury; KDIGO, Kidney Disease: Improving Global Outcomes; ECMO, extracorporeal membrane oxygenation.

Among the 83 recipients with culture-positive PF, 40 (40/83 = 48.2%) had postoperative pneumonia on Day 0. Twenty-eight (28/83 = 33.7%) recipients had postoperative pneumonia on Day 0 with at least one identical microorganism documented in both the PF and the donor respiratory sample.

The 30-day survival rate between recipients with postoperative pneumonia on Day 0 with at least one identical bacteria isolated from the PF compared to recipients without pneumonia on Day 0 or with pneumonia on Day 0 without identical bacteria isolated from PF was similar (85% vs. 86%, *p* = 0.90).

The 30-day survival rate between recipients with postoperative pneumonia on Day 0 with at least one identical bacteria isolated from PF compared to recipients with postoperative pneumonia on Day 0 without identical bacteria isolated from PF was similar (85% vs. 90.9%, *p* = 0.63).

Two (2/83; 2.4%) recipients had pleural empyema with at least one identical bacteria isolated from the PF (*Klebsiella pneumoniae* and *Corynebacterium striatum*, respectively), each occurring on day 8 post-transplant.

### Impact on Recipient Outcomes of Adequacy Between Antibiotic Prophylaxis and Antibiotic Susceptibility of Microorganisms Isolated in PF

Fifty-five (55/83; 66.3%) patients with culture-positive PF were treated with appropriate antibiotic prophylaxis initiated intraoperatively. Seventy-seven (77/83; 92.8%) patients with culture-positive PF received targeted antibiotic therapy after susceptibility testing with a standard duration of 7 days. The six patients who did not receive curative antibiotic therapy had culture-positive PF with oropharyngeal flora (*n* = 3), coagulase-negative staphylococci (*n* = 2), *Streptococcus anginosus* (*n* = 1) and *Proteus mirabilis* (*n* = 1). None of them had pneumonia on Day 0.

**FIGURE 2:**
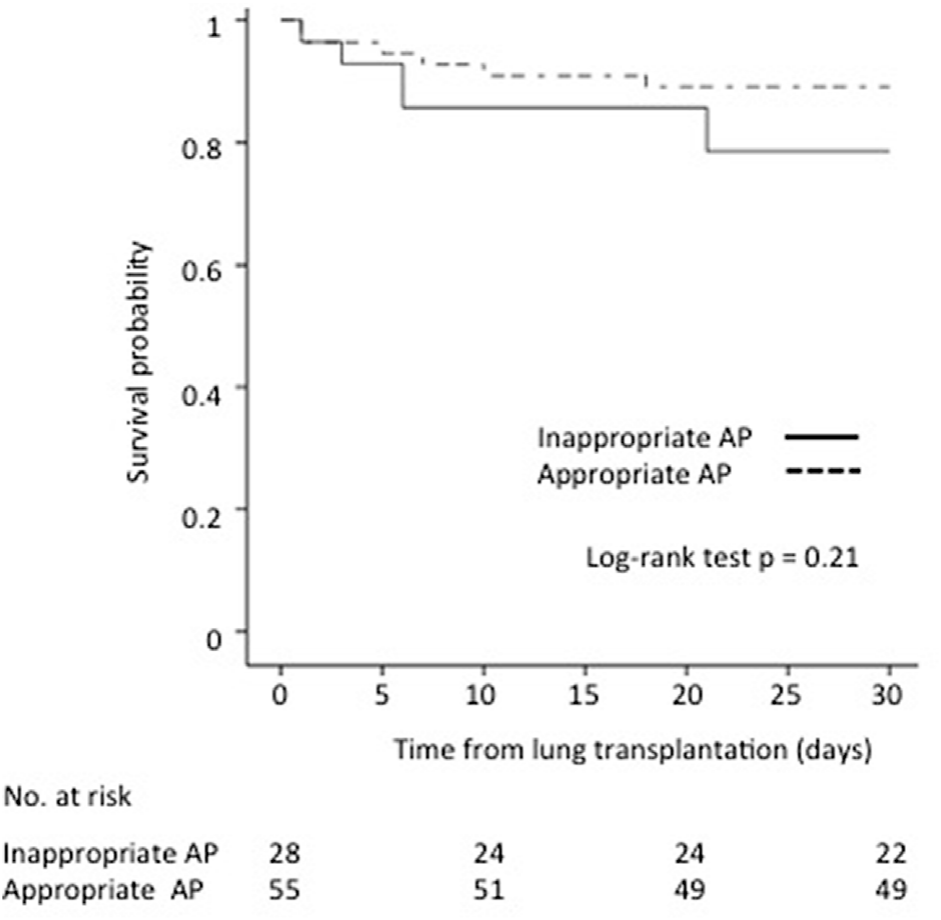
Impact of antibiotic prophylaxis (AP) adequacy on culture-positive PF on 30-day survival after lung transplantation.

The adequacy of antibiotic prophylaxis did not affect the 30-day survival of recipients with culture-positive PF compared to recipients without culture-positive PF (89.1% vs. 78.6%, *p* = 0.21, respectively) (Figure 2).

## Discussion

For the first time to the best of our knowledge, we designed a study to describe the impact of culture-positive PF on the outcomes of lung transplant patients. We reported a prevalence of 30% of recipients transplanted with grafts stored in culture-positive PF, which was associated with reduced 30-day survival. Although there is no consensual attitude to date, our results might argue for a systematic examination of the microbiological culture of the PF after LT.

The overall prevalence of culture-positive PF in solid organ transplantation is highly variable, having recently been reported as ranging from 37% in a systematic review (8) to 62.5% in a prospective study (23). Moreover, there are disparities between each organ. Only one study assessed the prevalence of culture-positive PF in LT that was 15% of 190 procedures (5), whereas it can reach 80% in renal transplantation (24) to almost 100% in liver transplantation (25).

The mechanism(s) responsible for the contamination of the normally sterile PF are hypothetical. The PF used in our centre is Perfadex^®^, which is a dextran-based solution that is low in potassium, reduces interstitial oedema and maintains the integrity of epithelial cells (26). Positive microbiological culture of the PF may include 1) endogenous dissemination of microorganisms contained in the organ during the storage, which could lead to a transplant with an already infected lung graft and/or to pleural empyema; or 2) extrinsic input during graft handling prior to transplant with a risk of secondary pleural empyema. According to our results, the high proportion of approximately 50% of postoperative pneumonia on Day 0, i.e., due to the same bacteria than those isolated in the PF, suggests endogenous contamination by passage of bacteria from the lung into the PF during storage.

The possible deleterious impact of culture-positive PF on early outcome with organ failure during the postoperative ICU stay and 30-day survival is threatening, especially in the face of its high prevalence. There is unresolved debate as to why culture-positive PF is associated with such detrimental outcomes. The largest prospective multicenter study on the impact of culturing PF on solid organ transplantation also reported nearly statistical significant association of culture-positive PF with recipient mortality (23). However, the authors acknowledged that there was no established explanation for this finding and that FP culture might be considered an overall indicator of transplant quality, including the donated organ and the transplant procedure. In our study, one third of recipients with culture-positive PF had postoperative pneumonia on Day 0 with at least one identical bacteria isolated from PF. In these cases, culture-positive PF may represent an indirect marker of donor lung infection. Nevertheless, the diagnosis of postoperative pneumonia on Day 0 after LT is highly challenging. The interpretation of postoperative chest X-ray is made difficult by the almost systematic presence of infiltrates, and the patient’s respiratory status is often uncertain. In addition, distinguishing with differential diagnoses such as primary graft dysfunction increases the difficulty. However, postoperative pneumonia on day 0 was diagnosed by considering international guidelines (13) and isolating bacteria at the infection threshold in bronchoalveolar lavage. Although donor-related infections have a disastrous impact on recipient outcomes (4, 25), the impact of postoperative pneumonia on Day 0 on mortality in lung transplant recipients remains unclear. We did not observe higher postoperative mortality among recipients with postoperative pneumonia on Day 0, weather or not associated to positive culture of PF.

Appropriate antibiotic prophylaxis against microorganisms isolated in the PF was administered to 60% of recipients. However, the adequacy of antibiotic prophylaxis did not influence the prognosis. One explanatory hypothesis is that 90% of transplant patients with culture-positive PF eventually received targeted antibiotic and/or antifungal therapy after identification and susceptibility testing of the microorganisms isolated from the PF within 48 h postoperatively.

Predicting and preventing the risk of culture-positive PF could help to reduce posttransplant morbidity and mortality rates. Disappointingly, we could not establish any risk factors for culture-positive PF from donor characteristics. Others identified advanced donor age as the main risk factor for culture-positive PF with high-risk microorganisms in solid organ transplants (23) and prolonged donor ICU stays (7). We showed that 70% of patients with culture-positive PF had at least one identical microorganism isolated from the donor respiratory specimen at the time of procurement. However, the time required for routine microbiological culture of the donor respiratory specimen is similar to that for PF. Given the worsening outcome when LT is performed with a graft stored in a culture-positive PF, special attention should be given to the diagnosis and treatment of donor pneumonia. This finding may also raise the issue of routine antibiotic prophylaxis administered to the donor to prevent pneumonia and possible contamination of PF. Although the identification of risk factors for culture-positive PF does not yet appear to be applicable in clinical practice, the use of rapid multiplex polymerase chain reaction (PCR) performed on PF could represent a promising diagnostic tool. This method allows rapid detection of bacteria, viruses and antibiotic resistance genes in a few hours (27–29) and improves antibiotic stewardship (30).

This study has some limitations, which are mainly inherent in its retrospective and single-centre design. Local centre policies on candidate selection and intra- and postoperative management complicate the external validity of the results. Our cohort suffers from a particularly high mortality rate in the postoperative period. However, we reported the largest series describing the microbiological features of PF in LT.

## Conclusion

Culture-positive PF has a high prevalence and may decrease lung transplant recipient survival. We advocate routine microbiological testing of the preservation fluid and treatment with targeted antibiotic therapy in case of positivity after lung transplantation. Further studies in LT are required to confirm these results and to improve understanding of the pathogenesis of culture-positive PF and its management.

## Data Availability

The raw data supporting the conclusion of this article will be made available by the authors, without undue reservation.
