# Supplementary material for: Impact of Culture-Positive Preservation Fluid on Early Morbidity and Mortality After Lung Transplantation
Source: Transpl Int. 2023 Feb 8;36:10826. doi: 10.3389/ti.2023.10826 (PMC9945515; doi:10.3389/ti.2023.10826)
Supplement: Supplementary file 1 [file Table1.docx]

**Table S1: Microorganisms isolated from donor respiratory samples**

| **Microorganisms (n = 119)** | **(n)** |
| --- | --- |
| **Gram-negative bacilli (n=51; 42.9%)** |  |
| *Enterobacter cloacae* | 10 |
| *Hafnia alvei* | 9 |
| *Escherichia coli* | 6 |
| *Pseudomonas aeruginosa* | 6 |
| *Klebsiella aerogenes* | 5 |
| *Serratia marcensens* | 4 |
| *Klebsiella pneumoniae* | 3 |
| *Morganella morganii* | 2 |
| *Citrobacter koseri* | 2 |
| *Haemophilus influenzae* | 2 |
| *Klebsiella oxytoca* | 1 |
| *Proteus mirabilis* | 1 |
| **Gram-positive cocci (n=47; 39.5%)** |  |
| *Staphylococcus aureus* | 40 |
| *Streptococcus pneumoniae* | 4 |
| *Streptococcus pyogenes* | 1 |
| *Streptococcus agalactiae* | 1 |
| *Streptococcus constellatus* | 1 |
| ***Others (n=21; 17.6%)*** |  |
| *Oropharyngeal flora* | 17 |
| *Branhamella catarrhalis* | 2 |
| *Corynebacterium striatum* | 1 |
| *Corynebacterium propinquum* | 1 |
