# Supplementary material for: Impact of Culture-Positive Preservation Fluid on Early Morbidity and Mortality After Lung Transplantation
Source: Transpl Int. 2023 Feb 8;36:10826. doi: 10.3389/ti.2023.10826 (PMC9945515; doi:10.3389/ti.2023.10826)
Supplement: Supplementary file 2 [file Table2.DOCX]

|  | **Recipients transplanted with culture-positive PF**  **(n = 83)** | **Recipients transplanted with culture-negative PF**  **(n = 188)** |
| --- | --- | --- |
| **Gram-negative bacilli (n)** |  |  |
| *Pseudomonas aeruginosa* | 21 | 66 |
| *Stenotrophomonas maltophilia* | 4 | 9 |
| *Acinetobacter baumanii* | - | 3 |
| *Achromobacter xylosoxidans* | 2 | 5 |
| *Hafnia alvei* | 5 | 8 |
| *Enterobacter cloacae* | 8 | 25 |
| *Serratia marcescens* | 5 | 3 |
| *Serratia urealytica* | 2 | - |
| *Klebsiella aerogenes* | 1 | - |
| *Morganella morganii* | 3 | 7 |
| *Citrobacter freundii* | - | 1 |
| *Citrobacter koseri* | 3 | - |
| *Klebsiella pneumoniae* | 8 | 25 |
| *Klebsiella varicola* | 2 | - |
| *Klebsiella oxytoca* | 1 | 1 |
| *Proteus mirabilis* | 3 | 5 |
| *Proteus vulgaris* | - | 4 |
| *Escherichia coli* | 14 | 19 |
| *Haemophilus influenzae* | 2 | 4 |
|  |  | - |
| **Oropharyngeal flora (n)** | 14 | 46 |
|  |  |  |
| **Gram-positive cocci (n)** |  |  |
| Methicillin-sensiitive *Staphylococcus aureus* | 23 | 36 |
| Methicillin-resistant *Staphylococcus aureus* | 2 | 4 |

**Table S2 : Bacteria isolated from pneumonia occurring during the ICU stay of patients transplanted with lung graft(s) stored in culture-positive or culture-negative preservation fluid**
